# Scottish and Newcastle Antiemetic Protocol (SNAP) 12-hour acetylcysteine regimen for paracetamol overdose reduces anaphylactoid reactions without compromising hepatic protection in all age groups: a secondary analysis

**DOI:** 10.1136/emermed-2024-214533

**Published:** 2025-08-24

**Authors:** Christopher Humphries, Janice Pettie, Bridget Agboola, Thomas M Caparrotta, Robert W Hunter, Emma Morrison, Euan A Sandilands, David J Webb, Michael Eddleston, James Dear

**Affiliations:** 1 The University of Edinburgh Centre for Cardiovascular Science, Edinburgh, UK; 2 The University of Edinburgh Centre for Precision Cell Therapy for the Liver, Edinburgh, UK; 3 National Poisons Information Service, Edinburgh, UK; 4 Bolton NHS Foundation Trust, Bolton, UK; 5 Edge Hill University, Ormskirk, UK

**Keywords:** overdose, poisoning, toxicology, pediatric emergency medicine, self-injurious behavior

## Abstract

**Background:**

Treatment with the 12-hour Scottish and Newcastle Antiemetic Protocol (SNAP) acetylcysteine regimen is associated with decreased length of stay and fewer anaphylactoid reactions in adult patients, and the protocol is now recommended by several UK organisations and used widely. One potential barrier to adoption is concern regarding the potential for variation in protocol performance with patient age. Anecdotally, this has led to slower adoption in paediatric settings.

**Methods:**

Secondary analysis of data from 2212 patients at the Royal Infirmary of Edinburgh, UK, treated with acetylcysteine for paracetamol overdose between 28 September 2013 and 27 September 2017. Patients were grouped into 10-year age ranges to allow comparison of treatment regimen performance across ages. Groups were compared for their rates of anaphylactoid reactions, duration of admission attributable to acetylcysteine infusion and severity of liver injury assessed by biochemical markers.

**Results:**

Patients in all age groups treated with SNAP experienced statistically significant reductions in anaphylactoid reactions. There were no significant differences in the severity of acute liver injury as assessed by biochemical results.

**Conclusion:**

This secondary analysis provides data to support the use of SNAP regardless of patient age and reassure clinicians that there is no evidence of previously unrecognised variation in protocol performance.

**WHAT IS ALREADY KNOWN ON THIS TOPIC**

**WHAT THIS STUDY ADDS**

- This retrospective analysis of audit data from 2212 patients provides evidence that all patients treated with SNAP experience statistically significant reductions in anaphylactoid reactions, with no difference in liver injury outcomes.

**HOW THIS STUDY MIGHT AFFECT RESEARCH, PRACTICE OR POLICY**

- This study provides additional evidence supporting the adoption of SNAP as the standard of care in the UK and other countries, and may help increase the adoption of SNAP in adolescent populations, where uptake has been slower.

## Introduction

 Paracetamol (acetaminophen) overdose presentations to UK emergency departments are estimated to exceed 100 000 a year.^1^ There is significant variation in the epidemiology of paracetamol overdose between patient age groups: approximately 38% of presentations occur in the 10–19 age group (which is defined as the period of adolescence by the WHO), but older patients are more likely to take larger overdoses.^1 2^ Theoretically, differences in the epidemiology of patient presentations or pre-morbid age-related factors could lead to unwarranted variation in the performance of antidote treatment protocols between age groups.

The only antidote in routine use for paracetamol toxicity is acetylcysteine (NAC). NAC restores hepatocyte glutathione levels, allowing detoxification of N-acetyl-p-benzoquinone imine—a product of paracetamol metabolism. Several NAC dosing regimens are in clinical use, but a common theme is that NAC (particularly at high blood concentrations) causes vomiting and anaphylactoid reactions in some patients. The original intravenous NAC regimen featured a loading dose of 150 mg/kg over 15 min. This was later extended to 60 min, as there was in vitro evidence of a dose-response relationship between NAC and histamine release, and it was felt that a decreased rate of infusion might lead to a decrease in the rate of dose-related adverse reactions.^3 4^

**Figure 1:**
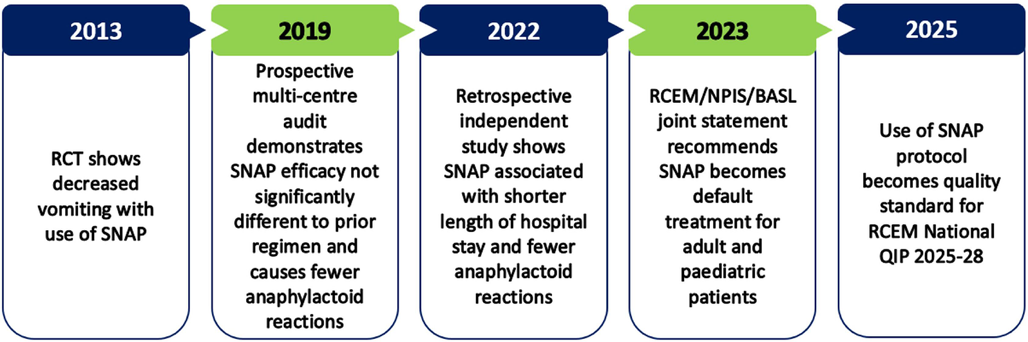
Timeline of evidence to support the use of the SNAP protocol in emergency departments. BASL, British Association for the Study of the Liver;NPIS, National Poisons Information Service; QIP, Quality Improvement Project; RCEM, Royal College of Emergency Medicine; RCT, randomised controlled trial; SNAP, Scottish and Newcastle Antiemetic Protocol.

A lower initial infusion rate protocol has now been developed, with the introduction of a novel 12-hour regimen (the Scottish and Newcastle Antiemetic Protocol—SNAP). SNAP delivers the same 300 mg/kg total dose of NAC in 12 hours, rather than the previously standard 21 hours. As shown in figure 1, SNAP has been demonstrated to reduce rates of vomiting and anaphylactoid reactions, decrease hospital length of stay and is not significantly different in its clinical safety across a range of performance measures.^5^,^7^ It is now recommended in the UK as the ‘default standard of practice in all emergency departments’, and the reported overall reduction in side-effects has allowed new clinical trials exploring higher NAC dosages.^8 9^

In early 2021, a survey of UK Emergency Department clinical leads (BA, unpublished data) was conducted, which demonstrated that (of 70 responding departments) 37% were using SNAP, and a further 39% were in the process of adopting it. It is unclear what impact the subsequent Royal College of Emergency Medicine (RCEM) guidelines may have had on adoption.^8 10 11^

Despite the momentum described in figure 1, to our knowledge, there are no disaggregated data allowing comparison of the performance of SNAP with the previous standard regimen across patient age groups, to examine whether specific age groups are experiencing unwarranted variation in regimen performance. The large prospective audit published in 2019 included patients aged 13 and older, but outcomes in adolescent and older adult subgroups have not been assessed.^6^

This lack of disaggregated data may be impacting the adoption of SNAP: based on conversations with colleagues across the UK, adoption of SNAP in paediatric settings which treat adolescent patients up to 16 years of age has been slower than for adults. This issue will soon become a particular focus for emergency departments, as the RCEM National Quality Improvement Project 2025–2028 will be assessing whether departments use SNAP as a metric of treatment quality in adolescent patients. The performance of the protocol in older adults has also not been evaluated previously.

The purpose of this secondary analysis was to explore the safety and effectiveness of the SNAP protocol across patient ages by reanalysing previously published Royal Infirmary of Edinburgh (RIE) data. Patients aged 13 and older are included, with patients subset into age groups, allowing examination of the data for variation within adolescent and adult population subgroups.

## Methods

### Design and setting

Secondary analysis of prospectively collected audit data from the RIE, UK, that were originally published in *EClinicalMedicine* in 2019.^6^ The introduction of SNAP reflects the only significant treatment change; there were no other changes to treatment guidelines during the data collection period.

### Participants

All patients aged 13 years and older admitted for treatment of paracetamol toxicity from 28 September 2013 to 27 September 2017, encompassing 2 years before (n=1075) and after (n=1137) the introduction of SNAP. During the data collection period, all patients aged 13 and older with paracetamol toxicity were cared for in RIE. Younger patients were cared for elsewhere and therefore did not have data available for analysis. Data from other hospitals in the original study were not included due to potential differences in care pathways across the adolescent population. Treatment for a single acute overdose within 24 hours of ingestion was based on a Rumack-Matthew ‘100-line’ nomogram.

The treatment regimens used and toxicity phenotypes (eg, staggered, therapeutic excess) are described in the original study.^6^

### Data collection

Data collection was undertaken prospectively using a pro forma paracetamol care pathway data collection form. This form provided required data for markers of liver injury and additional infusion requirements.

### Outcomes

The chosen safety outcome was the proportion of patients experiencing anaphylactoid reactions. Antihistamine prescriptions coinciding with NAC administration were used to estimate the rate of anaphylactoid reactions with both regimens. Efficacy outcomes were: proportion of patients requiring extended treatment beyond standard course, the mean hours attributable to infusion and the proportion of patients who reached predefined biochemical thresholds for liver injury, based on peak values during their admission. The number of cases of acute liver failure or death from acute liver failure was zero, and we therefore did not examine this outcome in our age-disaggregated analysis.

### Analysis

This secondary analysis was undertaken by an investigator independent of the original data collection. The investigator was not blinded to the final patient outcomes.

The primary aim of this study was to determine if the safety and effectiveness of the SNAP regimen is consistent across different age groups. Therefore, our analysis was designed to test for effect modification by age. We chose stratification by age cohort as the most direct and transparent method for this purpose. This approach allows for a straightforward comparison of the treatment regimen’s performance within each age group and avoids making assumptions about the functional form of the relationship between age and clinical outcomes (eg, linearity), which would be required in a multivariate regression model.

Adoption of SNAP in paediatric settings has been slower than in adult settings. Therefore, a subgroup comparison of adolescent patients was also performed, with the groupings designed to allow comparison between approximately the youngest 50% and oldest 50% of patients, to examine the consistency of SNAP performance across adolescence and ensure that differences in performance within the age range chosen were not masked. The results of this analysis are provided in online supplemental file 1.

Baseline characteristics, treatment required and markers of liver injury severity were collected, allowing comparison of protocol performance across ages and comparison of the relative performance of the two treatment regimens studied. The peak alanine aminotransferase (ALT) thresholds used were 150 (reflecting the ‘3 x upper limit of normal’ threshold used by the Food and Drug Administration in their assessment of hepatotoxic risk) and 1000 (reflecting the de facto standard for recognising hepatotoxicity in paracetamol toxicity).^12^,^14^

As laboratory results may be unavailable for clinical reasons (eg, due to discharge against medical advice, or non-completion of treatment), all results are given as ‘number of patients’, followed by the same number reflected as a percentage.

Within each age group, for each outcome of interest, Fisher’s exact test was used to compare the performance of the two treatment protocols using contingency tables in GraphPad Prism V.10.4.1. The calculation was two-sided, with 95% CIs calculated using the Newcombe/Wilson method with continuity correction. Statistical comparisons therefore examine differences attributable to the treatment regimen used within each age group, rather than differences between different age groups. To control for multiple comparisons for each outcome of interest (five age groups), a Bonferroni correction was applied to the standard α=0.05, resulting in an adjusted significance threshold of α=0.01. For the adolescent subgroup analysis, the Bonferroni correction resulted in a significance threshold of α=0.025. The same contingency tables were used to calculate the absolute risk reduction and 95% CIs for anaphylactoid reactions.

The ‘hours attributable to infusion’ data produced uses the same methodology as the previously published SNAPTIMED study—it is assumed that all patients receive the minimum standard regimen, and the mean number of extended infusion bags multiplied by the protocol duration of an extended infusion bag is added to this value.^7^

### Patient and public involvement

Patient and public involvement was not sought for this secondary analysis.

## Results

**Figure 2:**
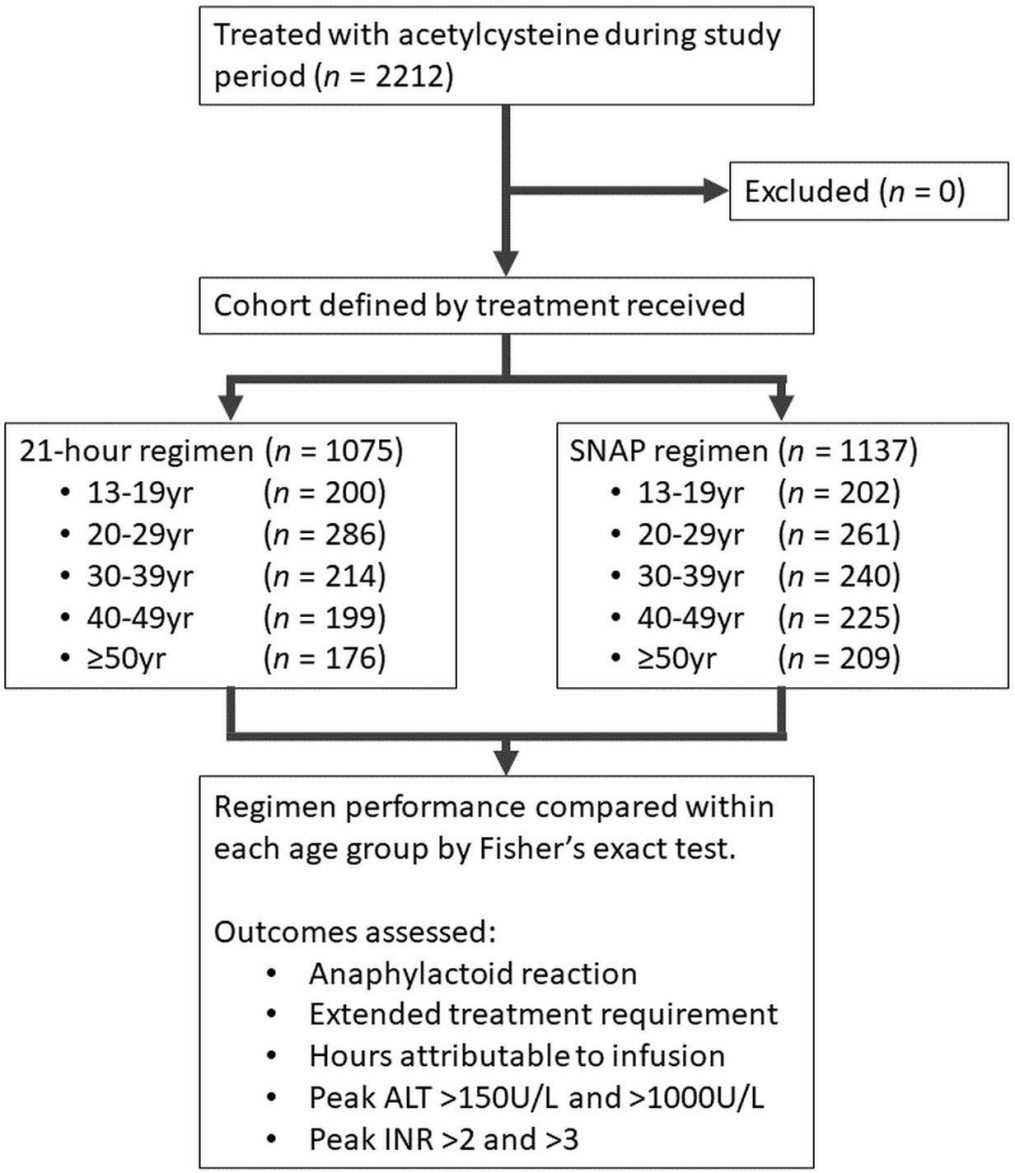
Study flow diagram. ALT, alanine aminotransferase; INR, international normalised ratio; SNAP, Scottish and Newcastle Antiemetic Protocol.

**Figure 3:**
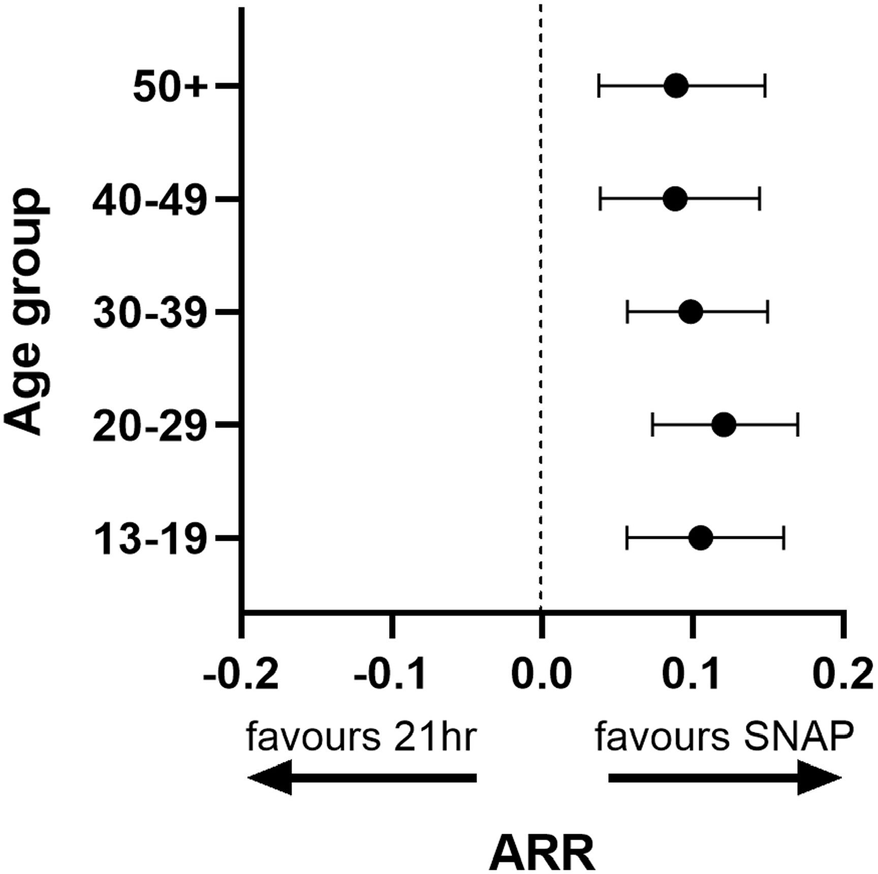
Forest plot of absolute risk reduction for anaphylactoid reactions with 95% CI tails. All age groups demonstrate statistically significant improvements in rates of anaphylactoid reaction with the SNAP regimen. ARR, absolute risk reduction; SNAP, Scottish and Newcastle Antiemetic Protocol.

We present all results stratified by age and treatment protocol. Baseline characteristics are presented in table 1, and a study flow diagram in figure 2. Information related to treatment course and injury severity is given in table 2. Statistical comparisons in table 2 are intra-age group comparisons of protocol performance (eg, proportion of 13–19 years old experiencing anaphylactoid reaction with 21 hours protocol vs SNAP). Statistically significant absolute risk reductions in rates of anaphylactoid reactions were seen for all groups and are displayed in figure 3.

**Table 1:** Baseline patient characteristics

		13–19	20–29	30–39	40–49	≥50
Number of patients (n, %)	21 hours	200, 19	286, 27	214, 20	199, 19	176, 16
	SNAP	202, 18	261, 23	240, 21	225, 20	209, 18
Age (median, IQR)	21 hours	17, 16–18	23, 21–25	35, 32–37	44, 41–47	56, 53–65
	SNAP	17, 16–18	24, 21–26	34, 31–36	43, 41–47	55, 52–63
Female (n, %)	21 hours	179, 90	209, 73	128, 60	126, 63	113, 64
	SNAP	179, 89	172, 66	176, 73	152, 68	127, 61
Dose ingested (median mg/kg, IQR)	21 hours	190, 144–267	169, 125–250	210, 141–298	221, 143–290	188, 117–279
	SNAP	203, 149–290	200, 136–290	166, 117–231	208, 132–311	177, 127–233
Overdose phenotype (n, %)						
<8 hour to NAC	21 hours	138, 69	123, 43	107, 51	98, 50	65, 37
	SNAP	122, 61	129, 50	87, 36	86, 38	96, 47
8–24 hour to NAC	21 hours	25, 13	55, 19	21, 10	29, 15	30, 17
	SNAP	34, 17	21, 8	17, 7	25, 11	29, 14
>24 hour to NAC	21 hours	6, 3	13, 5	5, 2	4, 2	8, 5
	SNAP	4, 2	5, 2	11, 5	5, 2	3, 1
Staggered	21 hours	19, 10	47, 16	49, 23	35, 18	27, 15
	SNAP	32, 16	56, 22	78, 33	78, 35	48, 23
Therapeutic excess	21 hours	12, 6	48, 17	29, 14	29, 15	46, 26
	SNAP	8, 4	49, 19	46, 19	31, 14	30, 15
ALT>ULN at presentation (n, %)	21 hours	12, 6	34, 12	39, 18	34, 17	43, 24
	SNAP	15, 7	46, 18	33, 14	49, 22	49, 23

Baseline patient characteristics for each age range. Data with unknown values excluded from population denominator.

Staggered overdose = deliberate overdose over more than 1 hour. Therapeutic excess = accidental overdose with the tablets taken for therapeutic indications.

ALT, alanine aminotransferase; NAC, acetylcysteine; SNAP, Scottish and Newcastle Antiemetic Protocol; ULN, upper limit of normal.

**Table 2:** Anaphylactoid reactions, infusion duration and injury severity by age group

		13–19	20–29	30–39	40–49	≥50
Anaphylactoid reaction (n, %)	21 hours	*23, 12*	*41, 14*	*22, 10*	*22, 11*	*19, 11*
	SNAP	*2, 1*	*6, 2*	*1, 0.4*	*5, 2*	*4, 2*
Extended treatment per protocol required beyond standard course (n, %)	21 hours	*21, 11*	*21, 7*	17, 8	*23, 12*	31, 18
	SNAP	*49, 24*	*57, 22*	36, 15	*49, 22*	43, 21
Mean total hours attributable to all infusions, all treated patients*	21 hours	22.8	22.6	22.6	23.6	25.2
	SNAP	17.3	16.3	15.1	16.5	15.9
Peak ALT >150 U/L (n, %)						
21 hours	13, 7	23, 8	22, 10	21, 11	29, 16	
SNAP	15, 7	21, 8	18, 8	26, 12	29, 14	
Peak ALT >1000 U/L (n, %)						
21 hours	5, 3	11, 4	9, 4	8, 4	14, 8	
SNAP	3, 1	11, 4	11, 5	9, 4	10, 5	
Peak INR >2 (n, %)						
21 hours	5, 3	5, 2	5, 2	9, 5	11, 6	
SNAP	3, 1	9, 3	8, 3	8, 4	9, 4	
Peak INR >3 (n, %)						
21 hours	3, 2	3, 1	4, 2	2, 1	4, 2	
SNAP	1, 0.4	3, 1	2, 1	6, 3	5, 2	

Data for each outcome of interest under each treatment protocol within each age group. Differences between the two treatment protocols were assessed within each age group for statistical significance. No comparisons were made between age brackets. Values with p<0.01 identified in *underlined italics*. Peak values as recorded at any time from presentation to discharge. Data with unknown values excluded from population denominator.

* Not assessed for statistical difference due to theoretical nature of data.

ALT, alanine aminotransferase; INR, international normalised ratio; SNAP, Scottish and Newcastle Antiemetic Protocol.

## Discussion

This study demonstrates that all patient age groups demonstrate statistically significant reductions in anaphylactoid reactions with the SNAP protocol, with no evidence to suggest differences in biochemical markers of liver injury. Where clinically important differences in protocol performance exist, they appear to be applicable across all age groups. Importantly, both the youngest and oldest patient age groups appear to receive the same benefits of shorter infusion duration, with no evidence to suggest that they are at increased risk of hepatotoxicity, liver failure or death.

The groups included in this subanalysis are well matched for comparative purposes. All patients aged 13 and older were cared for in RIE, ensuring that all potential patients were included and there were no differences in standard clinical care within the adolescent group.

The comparisons within younger and older adolescent patient subgroups in online supplemental table 1 demonstrated no concerning differences in protocol performance. Although slightly more patients in the younger SNAP group had a peak ALT >150 U/L, this group had a higher proportion of presentations beyond 8 hours, with ALT rises already in evidence. It is well established that treatment with NAC becomes less effective after 8 hours, and this difference in presentation pattern in our data is associated with a small increase in the number of patients experiencing higher ALT peak values.

The primary outcome of concern for paracetamol overdose remains acute liver failure, which currently lacks effective treatments other than liver transplantation.^15 16^ We were not able to provide a breakdown of outcome data by age, as outcome data were provided in an aggregated anonymised format for the original study. However, as the original study showed that the patients presented here had zero cases of liver failure and zero deaths from liver failure using either treatment protocol, readers can be reassured regarding each protocol’s performance.

Regarding the external validity of our findings, the UK uses a relatively conservative ‘100-line’ Rumack-Matthew nomogram and therefore has a lower threshold for treating patients after paracetamol ingestion than many other countries.^17^ This may have impacted the side-effect profile of NAC. We also acknowledge that this data set describes a preintervention and postintervention cohort, and there may be unidentified confounders within the study time period. This secondary analysis only sought to examine whether there are differences in protocol performance within age groups—future richer data sets have the potential to identify previously unrecognised subgroups with potential to benefit from personalised care.^18^ We acknowledge that our primary analysis is univariate within age strata and does not use multivariable regression to simultaneously adjust for all potential confounders. While our stratified approach directly addresses our research question regarding effect modification by age and is robust to model-based assumptions, we cannot exclude the possibility of residual confounding. However, given that baseline characteristics within each age group were largely comparable (table 1) and the magnitude of the benefit for anaphylactoid reactions was very large, it is unlikely that our conclusions are explained by minor imbalances in confounders.

In summary, it appears likely from these single-centre data that there are no clinically important differences between the safety and efficacy of SNAP and the 21 hours protocol within any of the patient age groups studied, with the exception of anaphylactoid reactions and hours attributable to infusion, which are improved in all ages with SNAP.

## Conclusion

SNAP has now been in clinical use for over a decade, but adoption in some patient groups has been held back by a lack of evidence to support change. This subanalysis provides data to support the widespread adoption of SNAP and reassure clinicians that the protocol is suitable for use in all patient groups. Our data demonstrate no evidence of difference in biochemical markers of injury and suggest that the benefits of SNAP (decreased anaphylactoid reactions and shorter inpatient admission) have the potential to benefit all patients, regardless of age.

## Supplementary material

10.1136/emermed-2024-214533 online supplemental file 1

## Data Availability

Data are available upon reasonable request.
