# Supplementary material for: Scottish and Newcastle Antiemetic Protocol (SNAP) 12-hour acetylcysteine regimen for paracetamol overdose reduces anaphylactoid reactions without compromising hepatic protection in all age groups: a secondary analysis
Source: Emerg Med J. 2025 Aug 24;43(1):e214533. doi: 10.1136/emermed-2024-214533 (PMC12772554; doi:10.1136/emermed-2024-214533)
Supplement: online supplemental file 1 [file emermed-43-1-s001.docx]

#### Supplemental table 1: Comparison of baseline characteristics and protocol performance in younger and older adolescent patients

A comparison of older versus younger adolescent patients (age >16, versus age ≤16) is provided in supplemental table 1. It demonstrates a consistency of protocol performance across adolescence and provides reassurance that use of this age range is appropriate and is not concealing differences in performance for the very youngest patients.

|  | |  | **13-16** | **17-19** |
| --- | --- | --- | --- | --- |
| **Number of patients (n)** | | **21hr** | 84 | 116 |
|  |  | **SNAP** | 96 | 106 |
| **Female (n, (%))** | | **21hr** | 77, 92% | 102, 88% |
|  |  | **SNAP** | 88, 92% | 91, 86% |
| **Dose ingested (median mg/kg, IQR)** | | **21hr** | 206, 148-272 | 180, 138-265 |
|  |  | **SNAP** | 209, 148-296 | 193, 149-288 |
| **Overdose phenotype (n, %)** | **<8h to NAC** | **21hr** | 59, 70% | 79, 68% |
|  |  | **SNAP** | 53, 55% | 69, 65% |
|  | **8-24h to NAC** | **21hr** | 10, 12% | 15, 13% |
|  |  | **SNAP** | 18, 19% | 16, 15% |
|  | **>24h to NAC** | **21hr** | 2, 2% | 4, 3% |
|  |  | **SNAP** | 2, 2% | 2, 2% |
|  | **Staggered** | **21hr** | 11, 13% | 8, 7% |
|  |  | **SNAP** | 20, 21% | 12, 11% |
|  | **Therapeutic excess** | **21hr** | 2, 2% | 10, 9% |
|  |  | **SNAP** | 2, 2% | 6, 6% |
| **ALT > ULN at presentation (n, %)** | | **21hr** | 5, 6% | 7, 6% |
|  |  | **SNAP** | 9, 9% | 6, 6% |
| **Adverse reaction: anaphylactoid reaction (n, %)** | | **21hr** | *7, 8%* | *16, 14%* |
|  |  | **SNAP** | *0, 0%* | *2, 2%* |
| **Extended treatment per protocol required beyond standard course (n, %)** | | **21hr** | 11, 13% | *10, 9%* |
|  |  | **SNAP** | 24, 25% | *25, 24%* |
| **Peak ALT >150U/L**  **(n, %)** | | **21hr** | 3, 4% | 10, 9% |
|  |  | **SNAP** | 9, 9% | 6, 6% |
| **Peak ALT >1000U/L**  **(n, %)** | | **21hr** | 1, 1% | 4, 3% |
|  |  | **SNAP** | 2, 2% | 1, 1% |
| **Peak INR >2**  **(n, %)** | | **21hr** | 2, 2% | 3, 3% |
|  |  | **SNAP** | 2, 2% | 1, 1% |
| **Peak INR >3**  **(n, %)** | | **21hr** | 1, 1% | 2, 2% |
|  |  | **SNAP** | 1, 1% | 0, 0% |

**Supplemental table 1. Baseline characteristics, safety and efficacy outcomes for younger vs. older adolescent patients. Baseline characteristics not assessed for differences. Safety and efficacy outcomes (i.e, adverse reactions and below) assessed for statistically significant differences. No comparisons were made between age brackets. Values with p<0.025 identified in *underlined italics*. Peak values as recorded at any time from presentation to discharge. Data with unknown values excluded from population denominator. *Not assessed for statistical difference due to theoretical nature of data. ALT: alanine aminotransferase. INR: international normalised ratio. NAC: acetylcysteine. ULN: upper limit of normal. Staggered overdose = deliberate overdose over more than 1h. Therapeutic excess = accidental overdose with the tablets taken for therapeutic indications.**
